# Concentrations of Pro-Inflammatory Cytokines Are Not Associated with Senescence Marker p16^INK4a^ or Predictive of Intracellular Emtricitabine/Tenofovir Metabolite and Endogenous Nucleotide Exposures in Adults with HIV Infection

**DOI:** 10.1371/journal.pone.0168709

**Published:** 2016-12-30

**Authors:** Brian M. Maas, Owen Francis, Katie R. Mollan, Cynthia Lee, Mackenzie L. Cottrell, Heather M. A. Prince, Craig Sykes, Christine Trezza, Chad Torrice, Nicole White, Stephanie Malone, Michael G. Hudgens, Norman E. Sharpless, Julie B. Dumond

**Affiliations:** 1 UNC Eshelman School of Pharmacy, University of North Carolina at Chapel Hill, Chapel Hill, NC, United States of America; 2 Gillings School of Global Public Health, University of North Carolina at Chapel Hill, Chapel Hill, NC, United States of America; 3 School of Medicine, University of North Carolina at Chapel Hill, Chapel Hill, NC, United States of America; 4 Lineberger Comprehensive Cancer Center, University of North Carolina at Chapel Hill, Chapel Hill, NC, United States of America; Rush University, UNITED STATES

## Abstract

**Objectives:**

As the HIV-infected population ages, the role of cellular senescence and inflammation on co-morbid conditions and pharmacotherapy is increasingly of interest. p16^INK4a^ expression, a marker for aging and senescence in T-cells, is associated with lower intracellular concentrations of endogenous nucleotides (EN) and nucleos(t)ide reverse transcriptase inhibitors (NRTIs). This study expands on these findings by determining whether inflammation is contributing to the association of p16^INK4a^ expression with intracellular metabolite (IM) exposure and endogenous nucleotide concentrations.

**Methods:**

Samples from 73 HIV-infected adults receiving daily tenofovir/emtricitabine (TFV/FTC) with either efavirenz (EFV) or atazanavir/ritonavir (ATV/r) were tested for p16^INK4a^ expression, and plasma cytokine and intracellular drug concentrations. Associations between p16^INK4a^ expression and cytokine concentrations were assessed using maximum likelihood methods, and elastic net regression was applied to assess whether cytokines were predictive of intracellular metabolite/endogenous nucleotide exposures.

**Results:**

Enrolled participants had a median age of 48 years (range 23–73). There were no significant associations between p16^INK4a^ expression and cytokines. Results of the elastic net regression showed weak relationships between IL-1Ra and FTC-triphosphate and deoxyadenosine triphosphate exposures, and MIP-1β, age and TFV-diphosphate exposures.

**Conclusions:**

In this clinical evaluation, we found no relationships between p16^INK4a^ expression and cytokines, or cytokines and intracellular nucleotide concentrations. While inflammation is known to play a role in this population, it is not a major contributor to the p16^INK4a^ association with decreased IM/EN exposures in these HIV-infected participants.

## Introduction

With improved antiretroviral (ARV) treatment, HIV infected patients experience increased life spans, and aging patients are susceptible to a host of new clinical challenges [1]. These challenges include increased risk for HIV-associated non-AIDS (HANA) conditions, including cardiovascular, renal, and hepatic disease. To date, treatment of HIV infection in older adults remains the same as that of younger adults, with additional comorbidity screening recommended as patients grow older. HIV-associated inflammation has been implicated in the early onset of many complications typically not seen until later years of life in uninfected individuals [2].

Tumor suppressor gene p16^INK4a^, is a marker of cellular senescence and biomarker for molecular age of several tissues, including T-cells[3]. HIV is thought to induce some functions of immunosenescence [2], which may contribute to the increased risk of age-related illness observed in infected patients. Although the exact mechanism is not well understood, inflammation may play a role. Compared to healthy individuals, patients with HIV infection have been shown to have increased levels of inflammatory cytokines [4, 5]. Through cyclin-dependent kinase pathways, p16^INK4a^ has been shown to regulate the production of proinflammatory cytokines such as IL-6 and IL-8 [6]. *In-vitro* work has shown that cells induced to senescence exhibit senescence-associated secretory phenotype (SASP), which is characterized by increases in GM-CSF, IL-6, -7, -8, -1β, -13, and GRO [7]. IFNγ decreases the formation of active metabolites of zidovudine and lamivudine in adipocytes and PBMCs [8], while TNFα has been shown to increase zidovudine metabolite formation. Several of these same cytokines are increased in HIV infection [9] and have been related to increased mortality in this population [10]. Defining the relationship between p16^INK4a^ and markers of inflammation in HIV infected adults will help better understand HIV-associated immune senescence [11]. Tenofovir disoproxil fumarate (TDF) and emtricitabine (FTC) are nucleos(t)ide reverse transcriptase inhibitors (NRTIs) and are recommended in first-line combination ARV therapies regardless of age. In order to exert their antiviral effect, they must cross the cellular membrane and undergo phosphorylation to their respective metabolites, tenofovir diphosphate (TFV-dp) and emtricitabine triphosphate (FTC-tp). We previously demonstrated that higher expression of p16^INK4a^ is associated with lower exposures of FTC-tp, dATP, and dCTP in HIV-infected participants [12]. Inflammation may contribute to this relationship, as it has been shown to regulate drug transporters, such as breast cancer resistance protein (BCRP) and P-glycoprotein 1(P-gp), on cell membranes [13] capable of effluxing NRTIs and their metabolites [14, 15]. Inflammation may also affect intracellular enzymes responsible for the metabolism of NRTIs [16]. While FTC is relatively non-toxic, kidney and bone toxicity with TFV are well-described adverse effects of the drug [17], and may potentially be related to drug concentrations [5, 18]. These toxicities are particularly critical in the aging population, as renal function and bone mass density decline occur naturally in this population, and TDF may compound these adverse effects.

Given these observations and potential mechanisms, we hypothesized that markers of inflammation may be related to p16^INK4a^ expression and the pharmacokinetics of NRTIs. Characterizing these relationships will help better understand the role of inflammation in HIV pathophysiology, with the ultimate goal of designing interventions to improve patient outcomes, such as prevalence of co-morbid conditions. The overall objective of this analysis is two-fold: 1) to determine whether markers of inflammation are associated with p16^INK4a^ expression, and 2) to determine if markers of inflammation are predictive of the observed IM/EN exposure in PBMCs.

## Methods

### Clinical Trial Design

A detailed description of the trial design and eligibility criteria has been previously published (5). In short, HIV-infected adults were recruited from UNC HealthCare Infectious Diseases Clinic (Chapel Hill, NC) and the Cone Health Regional Center for Infectious Diseases (Greensboro, NC). All participants received daily tenofovir disoproxil fumarate/FTC 300/200 mg with either efavirenz 600mg or atazanavir/ritonavir 300/200mg for at least 2 weeks, and provided four timed blood samples (predose, 2, 4–6, 10–14 hours post-dose).

The study protocol was approved by the Institutional Review Boards of both institutions (Clinicaltrials.gov NCT01180075), and conducted in accordance with all ethical standards, and in accordance with the principles of good clinical practice and all applicable regulatory requirements. The University of North Carolina at Chapel Hill Biomedical Institutional Review Board approved this study as IRB 09–2120. Its most recent approval after annual renewal (closed to enrollment, data analysis only) was July 7, 2016. Participants provided written consent, and went through a standard informed consent process involving: review of the IRB-approved consent form with trained study staff; time to review the consent form without study staff present; time to ask questions; and questioning by study staff determine that the participant understands the requirements of the study. Study staff witnessed the signing and dating the informed consent document and cosigned and dated the form; a copy was provided to the participant, a copy was placed in the medical record, and the original remained in the study chart. This standard operating procedure of the UNC Center for AIDS Research Clinical Pharmacology and Analytical Chemistry Laboratory was described in the IRB application and approved by the UNC Biomedical IRB for this study.

### Cytokines

At one of the pharmacokinetic time points, an additional blood sample was collected, centrifuged at 3000 RPMs for 10 minutes, and stored at -80°C for cytokine profiling. Measurement of 39 cytokine concentrations was performed in the Duke Regional Biocontainment Laboratory (RBL) Immunology Unit (Durham, NC) using MILLIPLEX® MAP Human Cytokine/Chemokine Premixed 39 Plex bead-based assay kit (EMD Millipore Corporation, Billerica, Massachusetts). Due to the large number of potential predictor variables, a subset of 16 cytokines that have been associated with HIV and aging was included in the analysis: TNFα, IFNγ, IL-1Ra, IL-6, IL-12P40, IL-12P70, IL-17α, MCP-1, MIP-1α, MIP-1β, MCP-3, MDC, GRO, sCD40L, fractalkine, and eotaxin [4, 19–21]. The lower limits of quantification for these cytokines were 3.23, 3.25, 17.87, 2.63, 3.9, 3.2, 3.21, 2.82, 3.36, 17.7, 14.3, 17.1, 19.6, 19.4, 79.0, and 4.15 pg/mL, respectively. The percentage of participants with detectable cytokine concentrations were: 100% TNFα, 89% IFNγ, 93% IL-1Ra, 49% IL-6, 64% IL-12P40, 92% IL-12P70, 64% IL-17α, 100% MCP-1, 67% MIP-1α, 93% MIP-1β, 56% MCP-3, 100% MDC, 100% GRO, 99% sCD40L, 93% fractalkine, and 100% eotaxin.

### Pharmacokinetics & p16^INK4a^ Expression

TFV-dp, FTC-tp, dATP, and dCTP concentrations in peripheral blood mononuclear cells (PBMCs) were measured using LC-MS/MS in the UNC Center for AIDS Research Clinical Pharmacology and Analytical Chemistry Laboratory [12]. Drug exposure was measured as area under the curve (AUC) using non-compartmental analysis in Phoenix WinNonlin 6.3 (Pharsight, A Certara Company, St. Louis, MO); the linear up/log down trapezoidal method [22] was used to calculate AUC over the dosing interval. Expression of p16^INK4a^ was determined using validated PCR-based methods [11] and final values were log_2_ transformed.

### Statistical Analysis

The goal of this analysis was to identify cytokines related to p16^INK4a^ expression and IM/EN AUCs. The causal direction of potential associations between p16^INK4A^ and cytokine concentrations is largely unknown, so unadjusted bidirectional association was tested. Because a large proportion of some cytokines were below the assay limit of detection, association with p16 ^INK4A^ was measured using a maximum likelihood approach that accounts for left censoring [23].

Cytokine concentrations were evaluated as potential predictors of IM/EN nucleotide AUCs using a linear model with elastic net selection, a penalized regression technique [24] for selecting variables that are most predictive amongst a large number of correlated potential predictors. Tuning parameters for the elastic net algorithm were chosen via 5-fold cross-validation with the optimal tuning parameter values chosen to minimize predicted residual sum of squares. To account for left-censored data, cytokines were dichotomized as detectable (1) or undetectable (0) if more than 20% of the concentrations were below the limit of detection, and cytokines detectable in 80% or more of participants were analyzed as continuous with results below the limit replaced with ½ the lower limit of quantification (LLQ). Chronological age in years was also included as a potential predictor variable.

Continuous cytokine concentrations were natural-log transformed and p16^INK4a^ measurements were log_2_ transformed. Data were analyzed in R 3.1.2 (r-project.org) and SAS version 9.4 using the GLMSELECT procedure and the NLMIXED procedure with code adapted from Nie and colleagues [25]. These analyses were exploratory in nature, thus no adjustment was made for multiple testing.

## Results

### Study Participants

The study enrolled 79 participants receiving TFV/FTC. Of those, 54 were receiving EFV and 25 were receiving ATV/r. The median (range) age was 48 years (23–73) and the median duration of HIV infection was 10 years. Though virologically suppressed during study participation, the historical median (range) CD4+ nadir was 253 (3–868) cells/mm^3^, with a peak viral load of 70,300 (50->1,000,000) copies/mL. Sixty-one percent of participants were African-American. Detailed demographics of the study population have been published [12]. One enrolled participant did not have a sample available for cytokine profiling. Of the remaining 78, five did not provide a sample for p16^INK4a^ measurement and six did not provide adequate samples for accurate calculation of IM/EN exposures. These individuals were excluded from analysis, resulting in 73 participants available for the p16^INK4a^ cytokine analysis, and 72 for the exposure-cytokine analysis.

### Correlation Analysis

**Fig 1:**
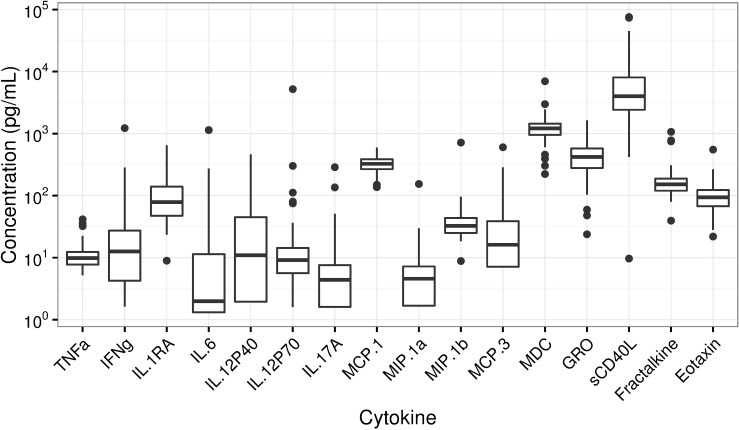
Boxplots of measured cytokine concentrations.

**Fig 2:**
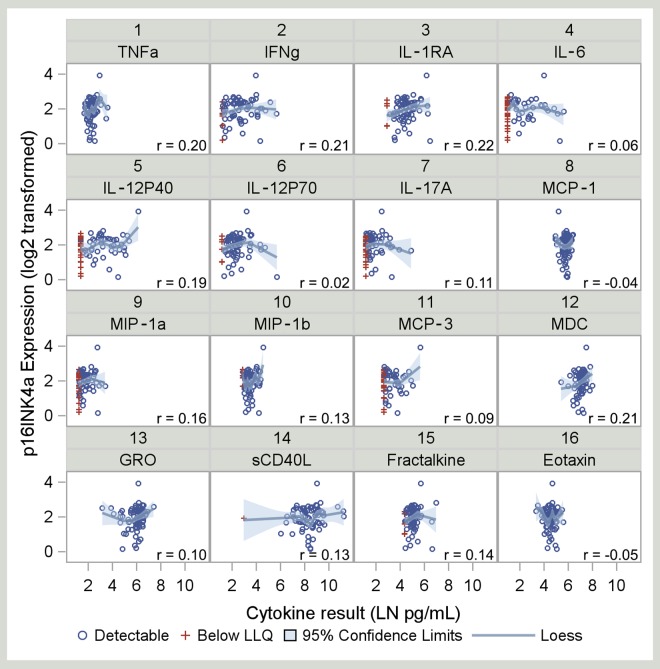
Scatterplots of p16^INK4a^ expression versus cytokine concentrations. Observations with quantifiable cytokine concentrations are displayed as blue circles; concentrations below the LLQ are displayed as red + symbols. Cytokine concentrations and p16^INK4a^ expression were natural-log transformed and log_2_ transformed, respectively. A descriptive LOESS curve is plotted handling below LLQ values as observed. Correlation estimates were less than or equal to 0.22 for all associations (Table 1). LLQ, lower limit of quantification.

A considerable number of cytokines were detectable (Table 1), but some remained below the LLQ. Five of the 16 cytokines had undetectable results in >20% of participants, six cytokines had undetectable results in 1–20% of participants, and five cytokines were 100% detectable. Fig 1 shows box plots of these cytokines; concentrations below the LLQ are included as half of the LLQ value for each cytokine. Table 1 shows the maximum likelihood correlation estimates and 95% confidence intervals for the unadjusted association between p16^INK4a^ and each cytokine. Correlation coefficient magnitudes ranged from 0.02 to0.22 and all 95% confidence intervals contained zero. The strongest associations were between p16^INK4a^ and interleukin-1 receptor agonist (IL-1Ra), interferon-gamma (IFNγ), macrophage-derived chemokine (MDC), and tumor necrosis factor alpha (TNFα; estimated correlation ≥0.2). Scatterplots of cytokine concentrations vs. p16^INK4a^ expression are shown in Fig 2.

**Table 1:** Correlation between natural-log transformed cytokines and log_2_ transformed p16^INK4a^ expression.

Cytokine	Proportion Detectable	Estimated Correlation (95% CI)
TNFα	1.00	0.20 (-0.02, 0.43)
IFNγ	0.89	0.21 (-0.02, 0.43)
IL-1Ra	0.93	0.22 (-0.01, 0.44)
IL-6	0.49	0.06 (-0.20, 0.32)
IL-12P40	0.64	0.19 (-0.05, 0.43)
IL-12P70	0.92	0.02 (-0.21, 0.26)
IL-17α	0.64	0.11 (-0.14, 0.35)
MCP-1	1.00	-0.04 (-0.27, 0.19)
MIP-1α	0.67	0.16 (-0.08, 0.40)
MIP-1β	0.93	0.13 (-0.10, 0.36)
MCP-3	0.56	0.09 (-0.16, 0.34)
MDC	1.00	0.21 (-0.01, 0.44)
GRO	1.00	0.10 (-0.13, 0.33)
sCD40L	0.99	0.13 (-0.10, 0.36)
Fractalkine	0.93	0.14 (-0.09, 0.37)
Eotaxin	1.00	-0.05 (-0.28, 0.19)

Proportion detectable denotes the proportion of participants with cytokine concentrations above the LLQ. The estimated correlation accounts for left-censored observations (below the LLQ); 95% confidence intervals for the correlation coefficient are provided. LLQ, lower limit of quantification.

### Elastic Net Analysis

For elastic net regression, five cytokines were dichotomized as detectable or undetectable and the remaining 11 were natural-log transformed. Results of the analysis are presented in Table 2. The R^2^ values are presented for each outcome and represent the proportion of variation explained by predictor variables in the model. The penalized parameter estimates for the selected variables describe the direction of prediction.

**Table 2:** Elastic net results for TFV-dp AUC, FTC-tp AUC, dATP AUC, and dCTP AUC

Outcome	n	r^2^	Predictor Variable	Parameter Estimate
FTC-tp AUC	72	0.0581	*Intercept*	12.22
			IL-1Ra	-0.1285
TFV-dp AUC	72	0.139	*Intercept*	7.941
			MIP-1β	-0.4483
			Age	0.03322
dATP AUC	72	0.0578	*Intercept*	8.499
			IL-1Ra	-0.07725
dCTP AUC	72	n/a	*No variables selected*	

For intracellular drug concentrations, higher concentrations of IL-1Ra were weakly predictive of lower FTC-tp exposures, and higher MIP-1β and younger age were predictive of lower TDF-dp exposures. Higher concentrations of IL-1Ra were also weakly predictive of lower pools of dATP, but no cytokines were predictive of endogenous dCTP pools.

## Discussion

Our earlier work identified an inverse relationship between p16^INK4a^ expression and IM/EN exposures. In an effort to explore these relationships further, we measured plasma cytokine concentrations in the same study participants. Correlation analysis was used to measure the association between p16^INK4a^ expression and cytokines concentrations. A considerable number of cytokines were below the limit of quantification for this analysis, which was accounted for using a maximum likelihood method for left-censored data. Overall, correlation between cytokines concentrations and p16^INK4a^ expression was weak. The magnitude of correlation coefficients ranged from 0.02 to 0.22 and each 95% CI covered the null value. IL-1Ra showed the strongest correlation with a coefficient of 0.22, followed by IFNγ and MDC with coefficients of 0.21 and TNFα with a coefficient of 0.20, but none were considered statistically significant. A stronger correlation was expected, as senescent cells typically exhibit SASP, which is characterized by increases in inflammatory cytokines. However, *in-vitro* work in human fibroblasts has demonstrated that SASP may be a result of DNA damage and not a direct consequence of p16^INK4a^ activation. The absence of any strong correlation in our analysis may be explained by Coppe and colleague’s suggestion that p16^INK4a^ positive cells may not harbor an *in-vivo* SASP [26].

From a pharmacology perspective, TFV, FTC, and their metabolites are known substrates of several drug transporters [14, 15], and the ENs are substrates of a number of nucleotide transporters [27]. Inflammation may influence the activity and expression of efflux transporters on PBMCs [13], leading to reduced drug concentrations. The activation state of the target cells can also affect the production of intracellular NRTI metabolites [14], and thus inflammatory processes may play a role in NRTI metabolism. However, we did not find that cytokines predicted IM/EN exposures. Although IL-1Ra and MIP-1β were chosen by the elastic net algorithm as significant predictors of FTC-tp, TFV-dp, and dATP exposures, they explained <14% of the variability in exposures.

Cytokines were largely detectable in this aging, HIV-infected population; however, some remained below the LLQ, which may bias our study results. The strength of correlations and amount of variability explained by our models was low. Cytokine concentrations and p16^INK4a^ expression in uninfected controls were not measured here for comparison of the overall state of inflammation in these virologically suppressed participants, though cytokine concentrations here are similar to others’ reported values for HIV-infected, suppressed patients[28], and our median IL-6 concentration is similar to the median concentration reported by Erlandson and colleagues in frail HIV-infected men (2.2 vs 2.3 pg/mL)[29]. For future studies, high-sensitivity ELISA should be considered for certain analytes, as multiplex assay can be imprecise at very low concentrations [30]. While likely unavoidable, it should also be noted that drug AUCs, and potentially inflammation, may be affected by increased adherence to the treatment regimen during the study period. Our results may also be due to the fact that monocytes are likely producers of pro-inflammatory cytokines in suppressed HIV-infected subjects, while p16^INK4a^ expression is measured in CD4+ T-cells. However, previous work indirectly supports a role of inflammation in p16^INK4a^ expression in HIV-infected patients not receiving ART with uncontrolled viremia.[3] This work showed that excess inflammation, assumed but not measured in these viremic patients, disrupted the typical relationship between chronologic age and p16^INK4a^ expression. In virologically suppressed, HIV-infected subjects, the typical relationship between the two was observed. This suggested a relationship between systemic inflammation, which we chose to measure with a broad panel of cytokines, and p16^INK4a^ expression, as a function of subject age.

Overall, we were unable to identify a relationship between cytokine concentrations and p16^INK4a^ expression, intracellular NRTI concentrations, or EN pools. This finding may underscore the complexities in cytokine signaling and suggest the need for different biomarkers as measures of inflammation to predict these outcomes. Cytokines, particularly IL-6 [31, 32], do predict clinical outcomes; for more subtle changes in pharmacology, other biomarkers of inflammation, such as soluble immune cell markers, cellular immune phenotypes, or kallistatin [33] may better predict changes that could influence drug toxicity and efficacy, and should be explored further.

## Supporting Information

S1 Table Minimal Dataset used for analysis. This table contains the data used in this analysis for each participant. Variables: ID, participant number; Age, in years; Gender; Ethnicity; Race; TxArm, EFV/FTC/TDF for efavirenz/emtricitabine/tenofovir disoproxil fumarate or ATV/r/FTC/TDF for atazanavir/ritonavir/emtricitabine/tenofovir disoproxil fumarate; Tobacco use; Alcohol use; CD4, screening visit CD4+ T-cell count, in cells/mm^3^; BMI, body mass index, in kg/m^2^; CrCL, calculated creatinine clearance, in mL/hr; Frailty Phenotype, positive or negative; Frailty Yes, number of positive frailty phenotype components; individual cytokine concentrations, in pg/mL; CD4:CD8 ratio; log_2_ p16^INK4a^ expression; individual regimen component calculated AUC (area under the curve) values for FTC (emtricitabine, in ng*hr/mL), ATV (atavanavir, in ng*hr/mL), EFV (efavirenz, in ng*hr/mL), RTV (ritonavir, in ng*hr/mL), TFV (tenofovir, in ng*hr/mL), FTCtp (emtricitabine triphosphate, in fmol*hr/million cells), TFVdp (tenofovir diphosphate, in fmol*hr/million cells), dATP (deoxyadenosine triphosphate, in fmol*hr/million cells), dCTP (deoxycytidine triphosphate, in fmol*hr/million cells), EFV_u (unbound efavirenz, in ng*hr/mL), ATV_u (unbound atazanavir, in ng*hr/mL), RTV_u (unbound ritonavir, in ng*hr/mL); unbound fraction (fu) values (as percentage) for EFV, ATV, and RTV.
(XLSX)
